# Genome-wide association studies using single-nucleotide polymorphisms versus haplotypes: an empirical comparison with data from the North American Rheumatoid Arthritis Consortium

**DOI:** 10.1186/1753-6561-3-s7-s35

**Published:** 2009-12-15

**Authors:** Heejung Shim, Hyonho Chun, Corinne D Engelman, Bret A Payseur

**Affiliations:** 1 Department of Statistics, University of Wisconsin-Madison, 1300 University Avenue, Madison, Wisconsin 53706 USA; 2 Department of Population Health Sciences, University of Wisconsin-Madison, 707 WARF Building, 610 North Walnut Street, Madison, Wisconsin 53726 USA; 3 Laboratory of Genetics, University of Wisconsin-Madison, 425-G Henry Mall, Madison, Wisconsin 53706 USA

## Abstract

The high genomic density of the single-nucleotide polymorphism (SNP) sets that are typically surveyed in genome-wide association studies (GWAS) now allows the application of haplotype-based methods. Although the choice of haplotype-based vs. individual-SNP approaches is expected to affect the results of association studies, few empirical comparisons of method performance have been reported on the genome-wide scale in the same set of individuals. To measure the relative ability of the two strategies to detect associations, we used a large dataset from the North American Rheumatoid Arthritis Consortium to: 1) partition the genome into haplotype blocks, 2) associate haplotypes with disease, and 3) compare the results with individual-SNP association mapping. Although some associations were shared across methods, each approach uniquely identified several strong candidate regions. Our results suggest that the application of both haplotype-based and individual-SNP testing to GWAS should be adopted as a routine procedure.

## Background

Advances in genotyping technology have stimulated genome-wide association studies (GWAS) of common human diseases. The high genomic density of single-nucleotide polymorphisms (SNPs) available on current genotyping platforms raises the prospect of combining neighboring SNPs into haplotypes for association analysis. Haplotype-based association testing offers several advantages over the standard "one-SNP-at-a-time" approach [1]. First, genome-wide haplotype approaches reduce the dimension of association testing when a single global test for a block is used. Performing fewer tests preserves power and helps to maintain reasonable false-positive rates. Second, haplotype methods facilitate detection of associations driven by *cis*-interactions among nearby SNPs that might be missed by methods that consider SNPs one at a time. Finally, haplotype approaches 1) recognize that variation in populations is inherently structured into genomic blocks and 2) exploit these correlations among SNPs. For all these reasons, using haplotypes in association testing is expected to increase power relative to single-SNP approaches, and studies based on human haplotype structure have provided support for this claim [2].

Nevertheless, haplotype association mapping faces several challenges. Haplotype block structure and phase is rarely observed in human genotyping data, requiring the application of statistical procedures that introduce additional error. Haplotype reconstruction methods that use different criteria produce different results [3], leaving the choice of the best approach for association mapping unclear. Moreover, when a block contains a large number of haplotypes, the increased degrees of freedom within a block can erode power.

The performance of haplotype-based and single-SNP association mapping have been compared, with mixed results. Power calculations using explicit analytical formula [4] and simulations based on the HapMap [2] suggested that using haplotypes can significantly improve power. Alternatively, Long and Langley [5] argued that single-SNP tests are at least as powerful in their simulation studies. Empirical comparisons have also yielded inconsistent conclusions [4].

The observed inconsistencies suggest that the performance of methods may depend on the nature of the data. Some associations might be detected more readily using individual SNPs, while others might only be discovered using haplotypes. Interestingly, most previous studies have restricted their comparisons of method performance to a small subset of the genome. To address these issues, we conducted GWAS with both haplotype-based and individual-SNP methods. By applying both approaches to the same genome-wide dataset (the North American Rheumatoid Arthritis Consortium (NARAC) provided by the Genetic Analysis Workshop 16 (GAW16)) we could directly compare the performance of these methods in an empirical context.

## Methods

### Phenotypes and genotypes

The data set from the NARAC contained 868 case subjects with rheumatoid arthritis (RA) and 1194 matched control subjects. Individuals were genotyped at 545,080 SNPs. Our study focused on the autosomes, where genotypes at 531,689 SNPs were available. After removing 17,754 SNPs that showed 1) a deviation from Hardy-Weinberg equilibrium in controls or 2) less than 0.001 minor allele frequency in the total sample, 513,935 SNPs were retained for further analysis.

### Haplotype block partitioning algorithms

Two methods were used to define haplotype blocks. The first method [6] was focused on D', a normalized measure of linkage disequilibrium (LD). This method identified informative pairs of SNPs as Category (1): those for which the upper 95% confidence bound on D' was between 0.7 and 0.98 and Category (2): those for which the upper confidence bound on D' was less than 0.9. We defined a haplotype block as a region where at least 95% of pairs among informative SNPs belonged to Category (1). The second method [7] used the four-gamete rule. For each pair of SNPs, the population frequencies of the four possible haplotypes were calculated, and the number of haplotypes with observed frequencies of at least 0.01 were counted. Blocks were constructed by combining consecutive pairs of SNPs for which only three haplotypes were observed. All methods were implemented in the computer program Haploview [8].

To examine the effects of changes in parameters on block definition, we varied the proportion of informative SNP pairs in Category (1) needed to combine blocks (in Gabriel's method) and the minimum frequency needed to call a haplotype "observed" (in the four-gamete rule algorithm). We compared the block sizes that resulted from applying these algorithms to the 8,051 SNPs on chromosome 21 and assuming a range of values for these two parameters. Although variation in block size was observed, block sizes for parameter values near the defaults were fairly stable; consequently, default values (0.95 in Gabriel's method, 0.01 in the four-gamete rule algorithm) were used in all subsequent analyses.

### Testing for associations between disease status and genotype

A high variance inflation factor (1.45) [9] suggested that association analyses of these data might be affected by population stratification. To account for these effects, we calculated top eigenvectors of the covariance matrix across the samples [10] using SNPs sampled every fifth position after excluding SNPs on the short arms of chromosomes 6 and 8, as Plenge et al. suggested [11]. Ten outliers, detected from ten eigenvectors, were excluded in all subsequent analyses. Both individual SNP associations and haplotype associations were measured by likelihood ratio tests via logistic regression where three eigenvectors were included as covariates to correct population stratification. These tests for individual SNP associations were implemented in the computer program PLINK [12,13]. For haplotype association tests, we estimated haplotypes in each block by the standard expectation maximization algorithm, implemented in PLINK, and conducted likelihood ratio tests via logistic regression with haplotypes by using the statistical package R [14]. Because we aimed to detect collective associations between groups of haplotypes and arthritis, we used a single global test of association for haplotypes. *p*-Values were compared to the Bonferroni threshold (alpha = 0.05/# tests) to identify statistically significant loci (SNPs not belonging to haplotypes were counted in both sets of analyses).

## Results

In what follows, we refer to a haplotype block as a partition containing at least two SNPs and a singleton as a partition containing only one SNP. GAB blocks and GAM blocks represent blocks constructed by the algorithm of Gabriel et al. [6] and the four gamete rule, respectively.

### Haplotype block partitioning

GAM partitioned the genome into more blocks (100,121) than GAB (97,881). On average, GAM blocks included more SNPs and were larger in size than GAB blocks (median number of SNPs: 3 in GAB; 4 in GAM), suggesting greater genomic coverage by GAM blocks. We note that a block is defined when a partition consists of at least two SNPs. Because the GAB method produces more singletons, the GAM method has both more blocks and a higher average block size. Block sizes estimated by both methods (GAM median size = 8,639 bp; GAB median size = 7,335 bp) were similar to those observed for other populations of European descent [15]. We also uncovered considerable variation in haplotype block structure across the genome, with block size ranging from 2 bp to 3,547,000 bp for both methods. Similar numbers of SNPs were assigned to haplotype blocks on the different chromosomes (median = 3 for most chromosomes). Because chromosomes vary in physical size, this suggests that variation in block size among chromosomes primarily reflected differences in the density of genotyped SNPs. Most (92%) SNPs were localized to GAB or GAM blocks, indicating that the density of genotyped SNPs was sufficient to conduct haplotype-based association analysis.

We ran Haploview on Intel Xeon 3 GHz dual Quad core system with 32 Gb of RAM. GAB block partitioning and GAM block partitioning on chromosome 22 including 8,205 SNPs required 19 and 14 minutes, respectively.

### Haplotype association test vs. individual SNP association test

A large number of tests on chromosome 6 showed strong associations (data not shown). The significance level, 0.05 becomes 9.73 × 10^-8 ^for single SNP, 2.71 × 10^-7 ^for GAB, 3.11 × 10^-7 ^for GAM after Bonferroni correction with total numbers of tests of 513,935 for single-SNP, 184,504 (97,881 GAB blocks plus 86,623 singletons) for GAB, 160,737 (100,121 GAM blocks plus 60,616 singletons) for GAM, respectively. Several associations survived the stringent Bonferroni correction for multiple testing (51 GAB blocks, 50 GAM blocks, and 21 individual SNPs) and some associations were shared among methods. A total of 8 out of 51 significant GAB blocks (Table 1) and 6 out of 50 significant GAM blocks included SNPs that were also significant in individual SNP association tests. Four SNPs showed significant associations using all three methods (Table 1). However, many associations were only detected when certain methods were applied. 43 GAB blocks and 44 GAM blocks that showed significant associations were not detected by individual SNP association tests and 11 of 21 SNPs that showed significant associations in individual tests were not significant in haplotype association tests.

**Table 1:** Significant associations detected by GAB algorithm

Chr	Start position	End position	*p*-value	Include significant SNPs^a^	Overlap with significant GAM blocks^b^
1	1,045,729	1,058,627	1.67 × 10^-14^	Yes	Yes
1	55,680,975	55,682,288	2.42 × 10^-8^	Yes	No
1	150,433,159	150,471,553	1.26 × 10^-8^	No	Yes
1	22,244,528	22,251,559	4.01 × 10^-10^	No	No
3	58,921,915	59,057,595	6.27 × 10^-11^	Yes	Yes
3	108,502,403	108,548,227	2.28 × 10^-11^	No	Yes
3	156,354,381	156,381,109	1.26 × 10^-7^	No	Yes
4	36,082,845	36,105,879	5.76 × 10^-8^	No	No
5	38,767,613	38,785,799	6.62 × 10^-8^	No	No
5	127,305,116	127,355,548	2.69 × 10^-7^	No	No
5	137,669,365	137,669,929	2.67 × 10^-8^	Yes	No
5	168,125,453	168,128,612	1.54 × 10^-7^	No	Yes
7	26,122,453	26,143,430	6.70 × 10^-9^	No	No
7	129,556,365	129,578,739	4.97 × 10^-12^	Yes	No
7	137,469,416	137,478,601	4.64 × 10^-8^	No	No
8	20,619,747	20,619,773	4.41 × 10^-8^	No	Yes
8	22,942,075	22,948,219	7.39 × 10^-10^	No	No
8	144,734,804	144,746,191	1.31 × 10^-8^	No	Yes
9	81,662,684	81,666,969	4.29 × 10^-8^	Yes	Yes
10	48,046,897	48,050,873	2.60 × 10^-11^	No	No
10	71,263,263	71,264,307	9.28 × 10^-11^	No	Yes
10	92,582,936	92,663,587	1.05 × 10^-37^	No	Yes
10	96,750,802	96,788,739	2.66 × 10^-11^	No	No
10	99,590,357	99,601,703	2.41 × 10^-7^	No	No
11	44,243,624	44,243,868	2.12 × 10^-10^	No	No
11	1,274,888	1,313,639	2.20 × 10^-9^	No	No
12	11,438,799	11,477,909	2.44 × 10^-7^	No	Yes
13	113,781,019	113,839,177	3.73 × 10^-12^	Yes	No
14	80,904,425	80,935,179	4.93 × 10^-9^	No	Yes
14	104,491,260	104,507,267	1.15 × 10^-9^	No	No
14	29,207,158	29,246,917	2.97 × 10^-10^	No	No
15	65,048,226	65,074,484	1.42 × 10^-12^	No	Yes
15	87,225,585	87,243,610	1.71 × 10^-12^	No	Yes
16	1,478,364	1,484,303	2.01 × 10^-8^	No	Yes
16	11,827,538	11,924,420	4.24 × 10^-10^	No	No
16	12,089,203	12,101,855	6.25 × 10^-10^	No	No
17	67,936,240	67,937,413	6.62 × 10^-9^	No	Yes
17	17,997,997	18,077,866	2.87 × 10^-9^	No	No
19	39,820,387	39,827,349	2.35 × 10^-8^	No	No
19	46,151,081	46,152,461	3.26 × 10^-8^	No	No
19	51,106,074	51,111,613	8.85 × 10^-8^	No	No
19	61,351,554	61,371,582	5.88 × 10^-11^	No	Yes
19	13,193,010	13,196,432	9.92 × 10^-11^	No	Yes
19	18,085,729	18,093,754	1.928 × 10^-7^	No	Yes
19	2,051,346	2,074,516	2.47 × 10^-7^	No	Yes
19	2,957,274	2,966,481	1.90 × 10^-11^	No	Yes
22	38,301,711	38,309,783	2.62 × 10^-7^	No	Yes
22	42,547,708	42,605,295	1.11 × 10^-13^	No	Yes
22	49,370,904	49,400,136	2.93 × 10^-8^	Yes	Yes
22	28,201,971	28,202,179	8.14 × 10^-12^	No	No
22	35,868,745	35,875,810	5.28 × 10^-8^	No	No

^a^Include SNPs which are significant in individual SNP association test

^b^Overlap with blocks which are constructed by four gamete rule and significant in a haplotype association test.

We asked whether reducing the significance threshold (9.73 × 10^-8 × ^2000) in individual SNP association tests and testing associations involving haplotypes within ± 100 kb of the resulting significant SNPs improved the consistency between individual SNP association tests and haplotype association tests. However, even using these extreme criteria, only 25 GAB blocks and 29 GAM blocks overlapped with the regions containing SNP association tests that were significant.

**Figure 1:**
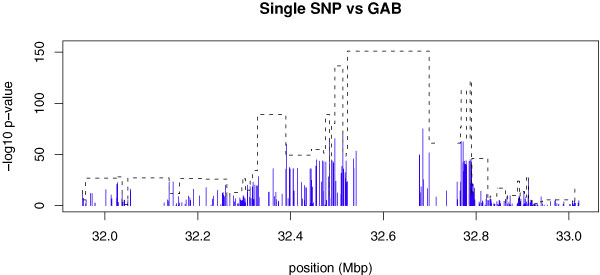
**A comparison of haplotype and individual association tests on HLA region**. -Log_10 _transformed *p*-values are plotted across chromosomal location 32 Mbp to 33 Mbp. The results from individual association tests are represented by solid blue sticks; the results from haplotype association tests with GAB blocks are represented by dotted black lines.

Most of the SNPs and haplotypes that showed significant associations on chromosome 6 were located near the HLA region. Similar to the pattern for significant associations in other genomic regions, *p*-values from haplotype association tests were smaller than *p*-values from individual SNP association tests (Figure 1).

Haplotype-based association tests of 1,578 blocks on chromosome 22 using R required 4 minutes on Intel Xeon 3 GHz dual Quad core system with 32 Gb of RAM.

### Haplotype association test: GAB vs. GAM

Twenty-five significant GAB blocks and 25 significant GAM blocks overlapped with each other. Interestingly, 18 GAB and GAM blocks out of 25 are identical, which is why these 18 haplotype-based associations using GAM and GAB blocks showed consistent results. For the remaining seven overlapped blocks, we hypothesize that signals of association with disease were strong, so that their detection was not sensitive to block partitioning. The fact that three of these blocks included SNPs that were significant in individual SNP association tests supports this hypothesis. In the regions where association tests in GAB and GAM blocks showed inconsistent results, we observed that differences between GAB block partitions and GAM block partitions were not unusual. In many cases, one block contained the other block with an additional one or two SNPs, but results from haplotype-based association tests in the two blocks were still substantially different.

## Conclusion

GWAS now commonly survey SNPs at a genomic density similar to this study. Consequently, our observation that most of the genome could be organized into multi-SNP haplotypes indicates that available resources are sufficient to conduct haplotype-based mapping on the genomic scale.

Those regions that were significantly associated with RA in both individual-SNP tests and haplotype-based tests represent promising candidates for further study. For example, the HLA region on chromosome 6 remained significant for all three genome-wide association tests, strongly suggesting that genes in this region contribute to disease risk.

Although some associations were observed consistently across methods, some associations were only detected using haplotype-based tests. Several factors might explain these differences. Haplotype-based methods required approximately 65% fewer tests than the individual-SNP approach. As a result, the multiple testing correction was less severe for haplotype-based methods. Haplotype-based methods can also detect *cis- *interactions among several causal variants [16]. Furthermore, because the power to detect associations is maximized when marker and causal variant frequencies are similar, analyses using haplotypes could find associations with rare alleles that analyses using individual SNPs may miss. We also discovered associations using individual SNPs that were not seen in haplotype tests. Perhaps these represented cases in which only a single SNP exhibited strong LD with a causal variant, so that forming haplotypes with several adjacent SNPs diluted the strength of association. Regardless of the explanation for observed differences among methods, our results indicate that the application of both individual-SNP and haplotype-based approaches to GWAS will maximize the potential for finding biologically important associations.

Although some regions show consistent significant associations in different block partitions (GAB and GAM), in most regions, haplotype-based association tests are really sensitive to changes in block partitions. This result suggests that the effects of other block partitioning algorithms on GWAS should be compared. For example, haplotype-based association testing using a sliding window of fixed physical or genetic size would be an alternative approach. Although this strategy is easily implemented, it ignores information about haplotype block structure. The variation in block structure across the genome suggests that methods that use this structure (such as those applied in this paper) should be more powerful for GWAS, but this issue needs to be examined.

Our study also suggests several avenues for future research. Additional measurements of the effects of different haplotype partitioning algorithms on the power of downstream association tests - in both simulations and empirical data - would be useful. For example, the error inherent in haplotype block estimation needs to be incorporated in association analysis. Furthermore, the likelihood ratio tests used here ignored the evolutionary relationships among haplotypes. An improved analysis that uses this information (e.g., a cladistic analysis [17]) would be worthwhile.

## List of abbreviations used

GAB: Blocks constructed by the algorithm of Gabriel et al. [6]; GAM: Blocks constructed by the four-gamete rule; GAW: Genetic Analysis Workshop; GWAS: Genome-wide association studies; LD: Linkage disequilibrium; NARAC: North American Rheumatoid Arthritis Consortium; RA: Rheumatoid arthritis; SNP: Single-nucleotide polymorphism

## Competing interests

The authors declare that they have no competing interests.

## Authors' contributions

HS and HC participated in the design of the study, performed the statistical analyses, and drafted the manuscript. CDE obtained IRB approval for the study, gained access to the dataset, participated in the design of the study, and helped revise the manuscript. BAP conceived of the study, participated in the design of the study, and helped draft and revise the manuscript. All authors read and approved the final manuscript.
